# The effect of respiratory viruses on immunogenicity and protection induced by a candidate universal influenza vaccine in mice

**DOI:** 10.1371/journal.pone.0215321

**Published:** 2019-04-15

**Authors:** Janelle Rowell, Chia-Yun Lo, Graeme E. Price, Julia A. Misplon, Roberta L. Crim, Priyanka Jayanti, Judy Beeler, Suzanne L. Epstein

**Affiliations:** 1 Office of Tissues and Advanced Therapies, US Food and Drug Administration, Silver Spring, Maryland, United States of America; 2 Office of Vaccines Research and Review, US Food and Drug Administration, Silver Spring, Maryland, United States of America; University of Iowa, UNITED STATES

## Abstract

Current approaches to influenza control rely on vaccines matched to viruses in circulation. Universal influenza vaccines would offer the advantage of providing broad protection against diverse strains of influenza virus. Candidate universal vaccines are developed using model systems, often testing in naïve animals. Yet the human population is not naïve, having varied immune histories that include exposure to viruses. We studied a candidate universal influenza vaccine (replication deficient adenoviruses expressing the conserved influenza A antigens NP and M2 [A/NP+M2-rAd]) given intranasally, the route previously shown to be most effective. To model recipients exposed to viruses, we used mice given rhinovirus (RV1B), respiratory syncytial virus (RSV-A2), influenza B virus, or influenza A virus before or after universal influenza vaccine. Vaccine performance was assessed by measuring immune responses to NP and M2, and monitoring weight loss and survival following influenza A challenge. Prior influenza A virus infection enhanced the response to the vaccine by priming to conserved influenza A antigens. RSV-A2 or RV1B had no effect on antibody responses to NP and M2 in serum. None of the viruses inhibited the ability of the vaccine to protect against influenza A virus challenge. The study demonstrates that the usefulness of this universal vaccine is not confined to the immunologically naïve and supports possible use in a human population with a varied history of respiratory infections.

## Introduction

Universal influenza vaccines have the potential to reduce the disease burden of seasonal and pandemic influenza. We have developed a candidate universal vaccine based on conserved influenza A virus (IAV) antigens nucleoprotein (A/NP) and matrix 2 (M2). Our previous studies demonstrated that DNA priming followed by boosting with a mixture of recombinant adenoviruses expressing A/NP and M2 (A/NP+M2-rAd) [1, 2] or a single intranasal dose of A/NP+M2-rAd [3, 4] protect naïve animals against subsequent IAV challenge of diverse strains and subtypes, preventing death and severe weight loss.

Preclinical testing of candidate vaccines in animal models typically uses naïve animals. However, vaccines for human use would be administered to individuals previously exposed to a wide range of antigens, including infections and other vaccines. In an effort to generate models that more closely recapitulate adult human immune responses, mouse models using a variety of prior immune stimuli have been developed [5, 6]. One study showed that sequential viral and parasitic infections alter the mouse immune system, resulting in responses more closely resembling those of adult humans [6]. Other work evaluating sequential infections has identified cross-protection between viruses, which is termed heterologous immunity [7]. In this scenario, T-cells primed by the first pathogen provide cross-protection against a subsequent differing pathogen; the cross-protection is not necessarily reciprocal [8]. In this way, sequential infections with various pathogens can alter the T-cell memory pool and increase or decrease subsequent responses to other pathogens [9, 10]. Prior infection history may also affect progression of disease caused by other viruses. For example, influenza virus infection protects mice against RSV-induced lung pathology [11], while latent infection with mouse herpesvirus-68 protects against IAV infection [12]. In some cases, instead of improving outcomes, a prior infection with one virus can lead to worse outcomes following infection with a second virus, despite contributing to clearance [9].

In humans, the influence of previous or ongoing infections on subsequent immune responses has been investigated for various viruses and other pathogens [13–15]. For instance, cytomegalovirus infection may influence immune responses to influenza [16]. Similarly, T-cell responses to influenza virus epitopes can overlap with reactivity to hepatitis C virus [17] or Epstein-Barr virus [18–20]. The sequence of exposure to multiple IAV infections may also influence immune responses and outcomes. Studies suggest immune imprinting occurs with the first influenza virus encountered [21–23], influencing susceptibility to different IAV subtypes seen later in life [24].

Responses to vaccines can also be influenced by prior infections. Infections initiated early in life may alter the response to subsequent vaccinations, possibly reducing the ability to respond to conventional vaccines [15, 25–27]. We previously demonstrated that vaccination history influences performance of our universal influenza vaccine in mice, resulting in enhancement or partial inhibition of universal vaccine-mediated protection, depending on the nature of the previous vaccines used [28]. Thus, it may be important to consider immune history when evaluating new vaccines.

In the human population, it would not be feasible to catalogue an individual’s every infection and then assess the impact on vaccination. It would also be difficult to model the lifelong sequence of viral infections, which is unique to each individual. However, the impact of previous infections can be studied in animal models using examples of common pathogens to provide a more realistic model than naïve animals alone. In the present study, we analyze the effects of acute respiratory viral infection on the performance of a universal influenza vaccine, including protection from IAV challenge and immune responses to vaccine antigens.

## Materials and methods

### Viruses

Human rhinovirus 1B, strain B632 (RV1B) was obtained from American Type Culture Collection (ATCC, Manassas, VA, USA). Virus was amplified and purified as previously described [29]. Briefly, RV1B was amplified in H1 HeLa cells (ATCC, CRL-1958). Cells were lysed by freeze-thaw, and then RV1B was precipitated using polyethylene glycol 6000. Virus was purified and concentrated using a centrifugal filtration device (Amicon Ultra 15 mL Filters (100,000 NMWL), MilliporeSigma, Burlington, MA). Fifty percent tissue-culture infectious dose (TCID_50_) was determined by titration in H1 HeLa cells. Respiratory syncytial virus, strain A2 (RSV-A2) was obtained from ATCC, then grown and prepared as previously described [30]. RV1B [31] and RSV-A2 [32] have been demonstrated to replicate in the respiratory tracts of mice.

Influenza A and B virus strains used were as follows: mouse-adapted A/Fort Monmouth/1/47 (H1N1) (A/FM) was provided by Earl Brown, University of Ottawa [33], A/Udorn/307/72 (H3N2) (A/Udorn) and B/Ann Arbor/1/86 (B/Ann Arbor) were obtained from Brian Murphy, National Institute of Allergy and Infectious Disease, National Institutes of Health. Viruses were prepared using embryonated hen’s eggs or lung homogenates of infected mice, as previously described [34].

### Recombinant adenoviral vaccines

Replication-deficient (E1 and E3 deleted) recombinant adenovirus-5 (rAd) vectors expressing conserved IAV antigens A/NP or M2 have been previously described [35, 36]. A recombinant adenoviral vector expressing influenza B virus nucleoprotein (B/NP-rAd) [36] was used as a specificity control because it confers no protection against IAV challenge.

### Mice

Female BALB/cAnNCR (BALB/c) mice were acquired from Charles River Laboratories (Ashburn, VA). Mice were 8–10 weeks of age at the beginning of experiments. All animal experiments were performed under ABSL-2 conditions in a facility accredited by the Association for Assessment and Accreditation of Laboratory Animal Care (AAALAC). Shelter, food and water were supplied in strict accordance with the recommendations in the Guide for the Care and Use of Laboratory Animals of the National Institutes of Health. Caging was in filter top microisolator caging with filtered air and ad libitum access to reverse osmosis filtered water and to LabDiet Iso pro 3000 Irradiated #25 feed (St. Louis, MO). Environmental enrichment was supplied to all animals. The protocol (protocol number 1991–06) was approved by the FDA White Oak Campus Animal Care and Use Committee. In influenza challenge experiments causing disease, analgesics were not used to avoid interference in immune responses, but distress was reduced by using a 25% weight loss humane endpoint, and any mice reaching that endpoint were euthanized. Animals were monitored daily with increased monitoring at a minimum of twice daily during challenge infections. Euthanasia before tissue harvest was by ketamine/xylazine overdose, while euthanasia due to body weight loss or termination of study was by carbon dioxide inhalation in a chamber where the carbon dioxide was from a cylinder source delivered by Euthanex equipment (Palmer, PA).

### Respiratory infections

For *in vivo* studies, mice received an intranasal dose of 10^4^ TCID_50_ A/Udorn or 10^5^ TCID_50_ B/Ann Arbor in 50 μL PBS; 5 x 10^5^ TCID_50_ RSV-A2 in 50 μL EMEM (Mediatech, Manassas, VA) containing 1% FBS, 100 mM MgSO_4_, and 50 mM HEPES [30]; 2 x 10^6^ TCID_50_ or 2 x 10^7^ TCID_50_ RV1B in 50 μL PBS. For RV1B, the two different doses both elicited immune responses in mice; the lower dose was used in all but one of the animal groups as noted in the text.

### Vaccination and challenge

Mice were immunized intranasally under isoflurane anesthesia with 10^10^ virus particles (vp) of B/NP-rAd, or with A/NP+M2-rAd (a mixture of 5 x10^9^ vp A/NP-rAd and 5 x10^9^ vp M2-rAd). Four weeks later mice were challenged with A/FM, using doses noted in figure legends, and monitored for body weight and survival. As mentioned above, 25% weight loss was used as a humane endpoint, and any mice reaching that endpoint were euthanized.

### T-cell responses to RV1B

Lung cells were re-stimulated with 10^6^ TCID_50_ RV1B and interferon-γ response was determined by enzyme-linked immunospot (ELISPOT) as described previously [35].

### Antibody responses to RSV-A2

Pre-immune sera and immune sera (three weeks following infection) were obtained from mice. Serum IgG antibodies to RSV nucleoprotein (RSV-N) were assessed by luciferase immunoprecipitation system (LIPS) using *Renilla* luciferase-tagged RSV-A2 nucleoprotein as previously described [37]. Serum samples from each animal experiment were tested in a single assay. The cutoff for a positive result was calculated for each assay based on 5 standard deviations above the mean value for pre-immune sera in that assay.

### Immune responses to influenza antigens

Three weeks after mice received A/NP+M2-rAd, lung and spleen cells from individual mice were assessed for IFN-γ production in response to peptides as follows: the dominant NP CD8 epitope in BALB/c mice NP_147-155_ (NP147), the consensus sequence of the M2 ectodomain M2e_2-24_ (M2e), and control SARS M_209-221_ (SARS) by ELISPOT as previously described [2, 35]. Sera were tested for IgG antibodies to influenza A/NP, B/NP, and M2e by ELISA using plates coated with M2e peptide or recombinant NP protein from strain A/PR/8/34 or B/Ann Arbor [1, 2].

### Statistical analysis

Statistical analyses were performed using SigmaPlot (Systat Software, San Jose, CA, USA). Survival data were analyzed by the log-rank test with pairwise comparisons using the Holm-Sidak test. Analysis of body weight following challenge was performed using One-Way ANOVA at a time-point (shown by an arrow in figures) when 100% survival was observed for all groups. Post hoc analyses used the Holm-Sidak method or Dunn’s method with the A/NP+M2-rAd group as the control. ELISPOT data were analyzed by two-way ANOVA or t-test, as stated in the figure legends. Multiple comparisons were made using the Holm-Sidak method with the A/NP+M2-rAd group as the pre-determined control for animal groups receiving different inocula, and SARS peptide as the control for different stimulating peptides within a single group of animals. P values less than 0.05 were considered statistically significant and are noted in the figures.

## Results

### Prior influenza A infection improves immune responses to A/NP+M2-rAd

The A/NP+M2-rAd universal vaccine candidate has previously been shown to provide broad cross-protection after a single dose. The best protection resulted from intranasal immunization, a route which generates mucosal T and B cell immunity efficiently [3] and is used currently in humans for live attenuated influenza vaccines. Vaccination to A/NP+M2 induces both antibody and T-cell responses, as reviewed in [38, 39]. Intranasal immunization induces antibodies and T cell responses, both systemically and locally in the respiratory tract. There are cytotoxic T lymphocytes in the lungs that can kill influenza virus-infected targets [40] and NP_147-155_-specific pentamer+ or tetramer+ CD8 T cells in the lungs [2, 3,40].

**Fig 1:**
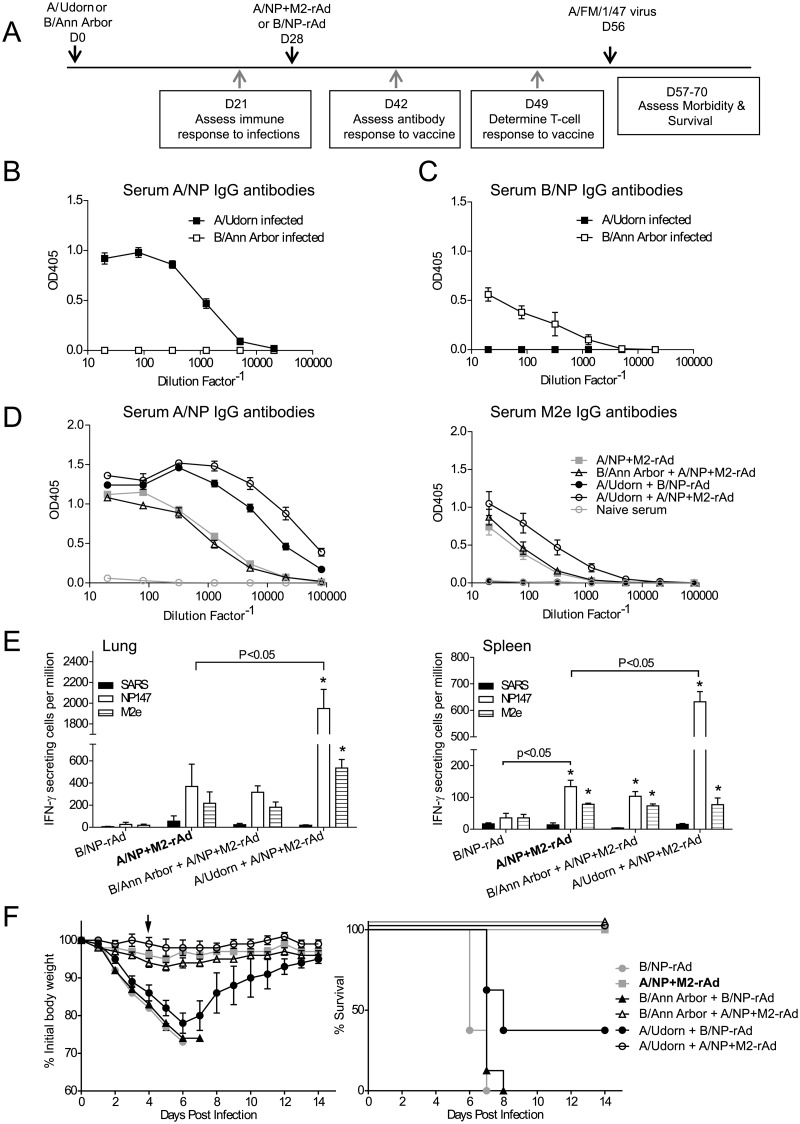
Infection with Influenza A or Influenza B prior to immunization with A/NP+M2-rAd. A) Study timeline provides details of experimental design. Sera collected 3 weeks after A/Udorn or B/Ann Arbor infection were tested for IgG antibodies by ELISA as described in Materials and Methods. B) Serum IgG antibodies to influenza A/NP. C) Serum IgG antibodies to influenza B/NP. n = 7 per group of mice infected with virus that matched the antigen on the plate, n = 3 for test of non-specific binding. D) Sera collected 2 weeks after A/NP+M2-rAd or B/NP-rAd were tested by ELISA as described in Materials and methods for IgG antibodies to influenza antigens A/NP or M2e. n = 10 per group. E) Three weeks after A/NP+M2-rAd or B/NP-rAd, lung and spleen cells from individual mice were assessed by ELISPOT for IFN-γ production in response to peptides NP147, M2e and SARS as specificity control. n = 2 for B/NP-rAd, n = 3 for all other groups. ELISPOT data were analyzed by two-way ANOVA using the Holm-Sidak method with A/NP+M2-rAd as control. A/NP+M2-rAd was predetermined as the comparator rather than comparing all pairs of groups, as explained in Materials and Methods. Significant differences between animal groups given different inocula are noted in the figure with horizontal brackets. Within an animal group, peptide comparisons are to SARS peptide as control and if significant indicated with asterisks* P<0.05 vs SARS. F) Left panel, percent initial body weight following challenge with 5.6 x 10^4^ TCID_50_ A/FM. Weight loss data were analyzed by One-Way ANOVA on the day indicated by the arrow. Post hoc testing was performed using the Holm-Sidak method to determine significant differences relative to A/NP+M2-rAd as the predetermined control group. The three groups B/NP-rAd, B/Ann Arbor+B/NP-rAd, and A/Udorn+B/NP-rAd all differed significantly from the A/NP+M2-rAd control group, P<0.001. Right panel, survival until death or 25% weight loss endpoint. n = 8 for B/NP-rAd, n = 10 for all other groups. Survival data were analyzed by log-rank test, using the Holm-Sidak method for pairwise comparisons. Regardless of prior infection, all groups receiving A/NP+M2-rAd were significantly different (P<0.05) from groups receiving B/NP-rAd, but not from other groups receiving A/NP+M2-rAd P<0.05 B/NP-rAd vs. B/Ann Arbor +B/NP-rAd, A/Udorn+B/NP-rAd.

We first studied the impact of prior influenza infection on subsequent performance of the universal vaccine candidate (Fig 1A). A/Udorn and B/Ann Arbor replicate well in mouse lungs [41], but do not induce significant clinical symptoms in mice. Three weeks later, mice had produced serum IgG antibodies specific to A/NP or B/NP (Fig 1B and 1C). Four weeks after infection, mice were immunized with A/NP+M2-rAd or B/NP-rAd intranasally, and an additional four weeks later challenged with influenza A/FM.

Serum IgG antibody responses to vaccine antigens A/NP and M2e were comparable between mice with no prior infection and mice with prior B/Ann Arbor infection, but enhanced in mice exposed to A/Udorn before receiving the universal vaccine (Fig 1D). Anti-A/NP but not anti-M2e antibody was induced by A/Udorn followed by B/NP-rAd. Consistent with elevated antibody responses, T-cell mediated immunity was enhanced in mice previously infected with A/Udorn as determined by IFN-γ ELISPOT (Fig 1E). In lung and spleen, T-cell responses to NP147 were approximately 5-fold greater in mice with a history of A/Udorn infection compared to mice with no prior infection, while responses to M2e were not elevated. In contrast, mice previously exposed to B/Ann Arbor had T-cell responses to NP147 and M2e in lung and spleen similar to those observed in mice with no previous exposure.

Weight loss and survival curves are shown in Fig 1F. In control mice with no prior infection history, A/NP+M2-rAd alone protected from severe influenza disease, with 100% survival and minimal weight loss. Thus for survival as an endpoint, only inhibition but not enhancement can be assessed under these conditions. However, either enhancement or inhibition of vaccine-specific immune responses and weight loss would be detectable. Mice previously infected with A/Udorn followed by A/NP+M2-rAd immunization had minimal weight loss following challenge and 100% survived, an outcome as good as A/NP+M2-rAd in naïve mice. Infection with A/Udorn without the universal vaccine (A/Udorn followed by B/NP-rAd control) provided modest cross-protective immunity against the mismatched A/FM challenge virus. Mice exhibited significant weight loss and 40% survival. Prior exposure to B/Ann Arbor did not significantly affect outcomes of vaccination followed by influenza A challenge. Mice exposed to B/Ann Arbor and then A/NP+M2-rAd exhibited minimal weight loss following challenge, comparable to mice with no prior exposure, and all mice survived IAV challenge. Mice given B/Ann Arbor and the control immunization, B/NP-rAd, lost significant weight following A/FM challenge and all mice succumbed.

### RSV-A2 or RV1B viruses do not affect vaccine protection

**Fig 2:**
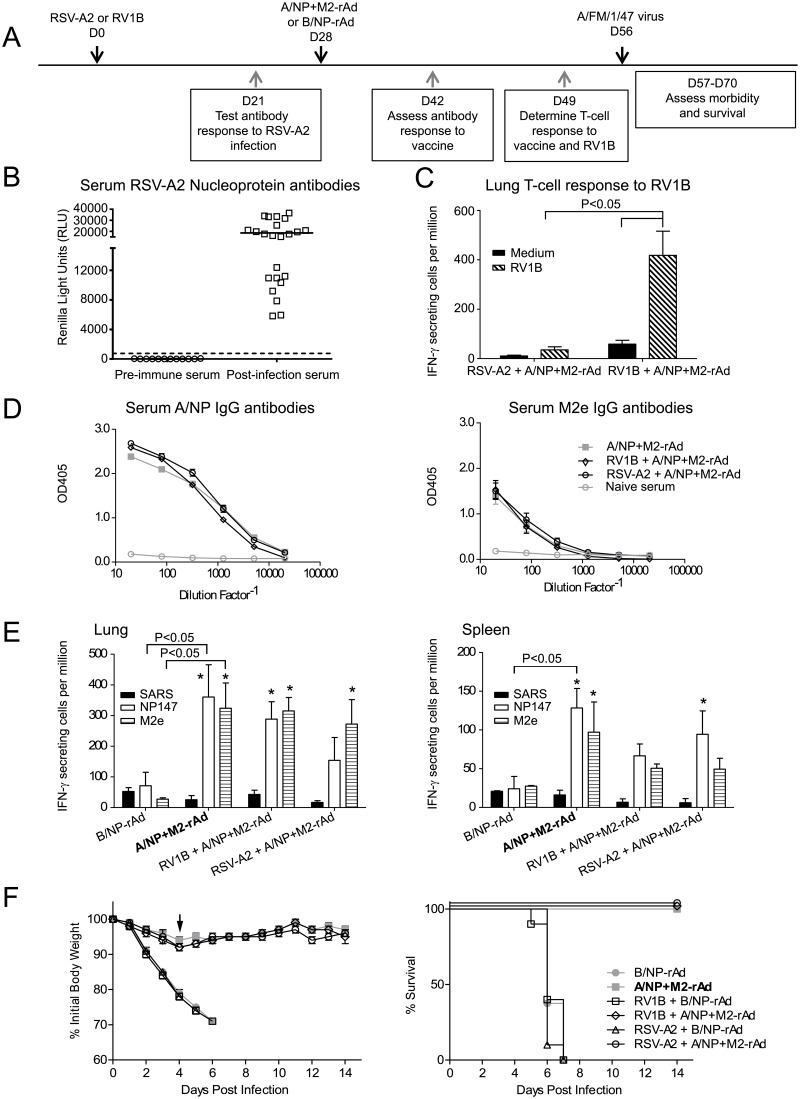
Infection with RV1B or RSV does not affect performance of A/NP+M2-rAd vaccine. A) Study timeline. B) Serum antibodies of individual mice to RSV-A2-N were assessed by LIPS assay. The cut-off for a positive result is noted by the dotted line above the X-axis. C) T cell response of individual mice to RV1B infection was assessed by ELISPOT. Whole purified RV1B virus was used for re-stimulation. n = 3. Significant differences were determined by Two-Way ANOVA using the Holm-Sidak method for post hoc testing of pairwise comparisons. D) Serum IgG antibodies to influenza antigens A/NP and M2e were assessed by ELISA as in Fig 1. E) Three weeks after A/NP+M2-rAd or B/NP-rAd, IFN-γ ELISPOT was performed as in Fig 1 using cells from lung (left) and spleen (right) stimulated by the indicated peptides. n = 2 for B/NP-rAd, n = 3 for all other groups. Significant differences between animal groups given different inocula were determined by two-way ANOVA using the Holm-Sidak method for multiple comparisons, with A/NP+M2-rAd as predetermined control group for comparisons. Significant differences between groups are noted in the figure with horizontal brackets. Within an animal group, peptide comparisons are to SARS peptide as control and if significant indicated with asterisks. * indicates P<0.05 vs. SARS. F) Weight loss (left) and survival (right) are shown following influenza challenge with A/FM. The challenge dose was 9.4 x 10^4^ TCID_50_ due to a technical issue. n = 8 for B/NP-rAd immunized groups, n = 10 for A/NP+M2-rAd immunized groups. Weight loss data were analyzed on the day indicated by the arrow by One-Way ANOVA on the ranks using Dunn’s method to compare other groups with the A/NP+M2-rAd group as predetermined control. The three groups RSV-A2 + B/NP-rAd, RV1B + B/NP-rAd, and B/NP-rAd all differed significantly from the A/NP+M2-rAd group, P<0.001. Survival data were analyzed by log-rank test using the Holm-Sidak method for pairwise comparisons. Regardless of prior infection, all groups receiving A/NP+M2-rAd were significantly different (P<0.05) from groups receiving B/NP-rAd, but not from other groups receiving A/NP+M2-rAd.

We next examined the effect of two common respiratory pathogens, RSV-A2 and RV1B, on the performance of the universal vaccine. One month prior to immunization with A/NP+M2-rAd, mice received intranasal inoculations of 5 x 10^5^ TCID_50_ RSV-A2 or 2 x 10^7^ TCID_50_ RV1B (Fig 2A). These viruses have previously been shown to replicate in mouse lungs [31, 42]. To confirm exposure, immune responses to each virus were assessed. In mice exposed to RSV-A2, we detected serum antibodies to RSV-A2 nucleoprotein by LIPS, but none in pre-immune serum (Fig 2B). Infection with RV1B caused increased frequency of IFN-γ secreting lung cells in response to RV1B virus in comparison to cells from RSV-A2-infected mice (Fig 2C). Four weeks after viral exposure, mice received A/NP+M2-rAd or B/NP-rAd. Immune responses to NP and M2 were not altered by prior exposure to RSV-A2 or RV1B, as determined by measures of serum antibodies and IFN-γ production by lung and spleen cells (Fig 2D and 2E). Four weeks after rAd, mice were challenged with influenza A/FM. As shown in Fig 2F, mice given A/NP+M2-rAd were protected from substantial weight loss and 100% survived influenza A challenge, with no effect of prior exposure to RSV-A2 or RV1B. Mice immunized with the B/NP-rAd control, with or without prior RSV-A2 or RV1B, lost a significant percentage of body weight and none survived challenge.

**Fig 3:**
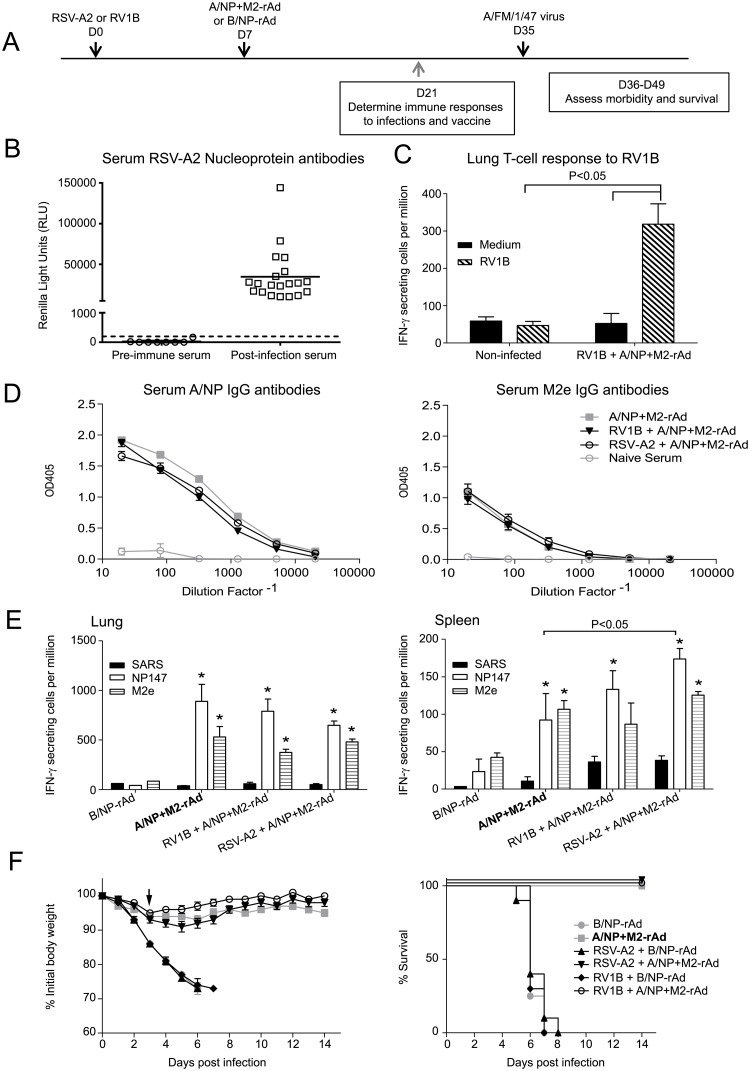
Recent infection with RV1B or RSV-A2 does not inhibit the performance of universal vaccine. A) Study timeline. B) RSV-N IgG antibodies measured in individual sera by LIPS assay. The dotted line indicates the cut-off for a positive result. C) IFN-γ T-cell response to RV1B virus in the lungs of individual mice (n = 3/group) measured by ELISPOT. Significant differences were determined by two-way ANOVA using the Holm-Sidak method for post hoc testing of pairwise comparisons. D) Serum antibody responses to A/NP (left) and to M2e (right) were assessed by ELISA. n = 10 per group. E) IFN-γ ELISPOT was performed as in Fig 1 stimulated by the indicated peptides, using lung (left) and spleen (right) cells. n = 1 for B/NP-rAd lung, n = 2 for B/NP-rAd spleen, n = 3 for all other groups. Significant differences between groups were determined by two-way ANOVA using the Holm-Sidak method with A/NP+M2-rAd as the predetermined control group for post hoc testing. Significant differences between groups are noted in the figure with horizontal brackets. Within an animal group, peptide comparisons are to SARS peptide as control and if significant indicated with asterisks, * P<0.05 vs SARS. F) Weight loss (left) and survival (right) following influenza challenge with 5.6 x 10^4^ TCID_50_ A/FM. n = 8 for B/NP-rAd immunized mice, n = 10 for A/NP+M2-rAd immunized groups. Weight loss data were analyzed on the day indicated by the arrow by One-Way ANOVA using the Holm-Sidak method, with the A/NP+M2-rAd group as predetermined control for post hoc comparisons. The RSV-A2 + B/NP-rAd, RV1B + B/NP-rAd, and B/NP-rAd groups all differed from the A/NP+M2-rAd control group, P<0.001. Survival data were analyzed by log-rank test with the Holm-Sidak method for pairwise comparisons. Regardless of prior infection, all groups receiving A/NP+M2-rAd were significantly different (P<0.05) from groups receiving B/NP-rAd, but not from other groups receiving A/NP+M2-rAd.

The timing of respiratory infection could influence vaccine performance. To testrecent infection, mice were given 5 x 10^5^ TCID_50_ RSV-A2 or 2 x 10^6^ TCID_50_ RV1B and immunized with A/NP+M2-rAd or B/NP-rAd one week later (Fig 3A). Immune responses to RSV-A2 or RV1B, NP and M2e were measured in samples collected 2 weeks after vaccination. Antibodies to RSV-A2 N and T-cell responses to RV1B were detected in the respective groups (Fig 3B and 3C). Serum antibody responses to vaccine antigens did not differ between the groups (Fig 3D). Likewise, groups with and without prior infection had comparable frequencies of IFN-γ secreting lung cells stimulated by vaccine antigens, with no statistically significant differences (Fig 3E). In the spleen, there was an increased frequency of IFN-γ secreting cells responsive to NP147 in the group that received RSV-A2 compared to mice given A/NP+M2-rAd only. However, this increase was not significant if comparisons were made after subtracting the nonspecific response to SARS peptide. The baseline frequency of IFN-γ secreting spleen cells (SARS control) in RSV-A2 and RV1B groups was greater than for A/NP+M2-rAd only, though not significantly. One month after receiving A/NP+M2-rAd or B/NP-rAd, mice were challenged with A/FM. Mice given A/NP+M2-rAd were protected from severe weight loss and death following A/FM challenge, despite a history of recent respiratory infection (Fig 3F). Weight loss was not significantly different for mice given A/NP+M2-rAd alone or after RV1B or RSV-A2 infections.

**Fig 4:**
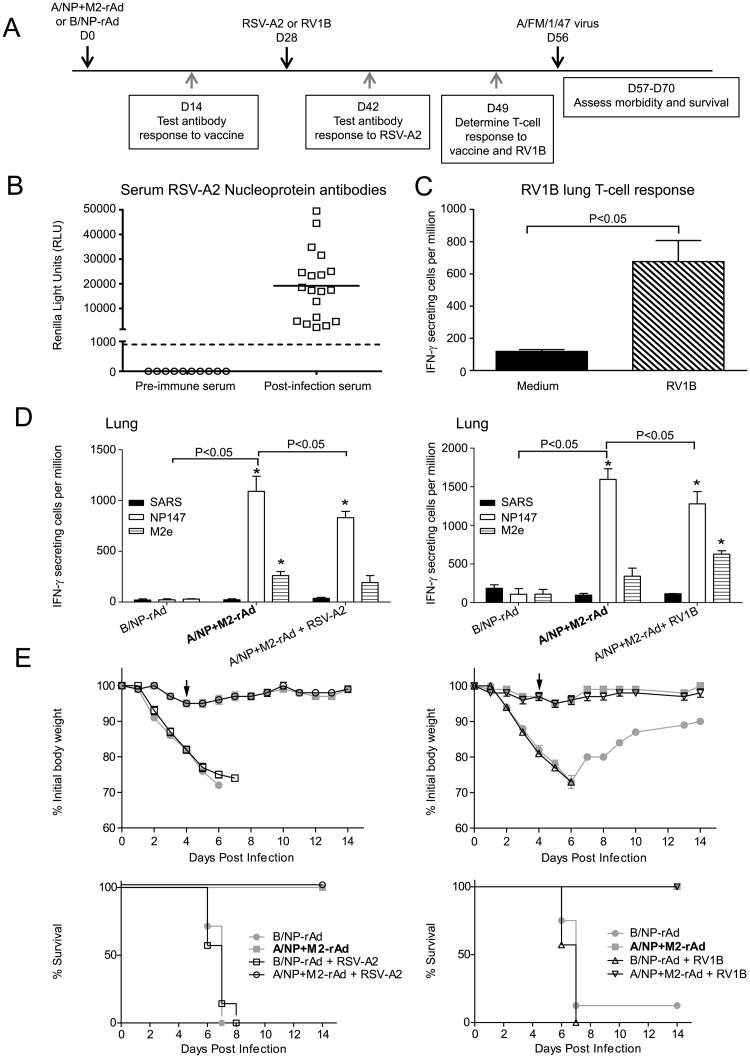
Intervening infection with RV1B or RSV-A2 does not affect universal vaccine protection. A) Study timeline. B) Intervening RSV-A2 infection was confirmed by antibody responses to RSV-N by LIPS assay as in Fig 2. In this test, the cut-off for a positive result is indicated by the dotted line above the X-axis. C) Intervening RV1B infection was confirmed by T-cell responses assessed by ELISPOT as in Fig 2. n = 3. Significant difference was determined by t-test. D) T cell response to influenza antigens was assessed following rAd vaccination using lung cells from mice infected with RSV-A2 (left panel) or RV1B (right panel) tested by IFN-γ ELISPOT. n = 2 for B/NP-rAd, n = 3 for all other groups. Significant differences between groups were determined by two-way ANOVA using the Holm-Sidak method, with A/NP+M2-rAd as predetermined control for post hoc testing. Significant differences between groups are noted in the figure with horizontal brackets. Within an animal group, peptide comparisons are to SARS peptide as control, and if significant indicated with asterisks, * P<0.05 vs SARS E) Weight loss (top graphs) and survival (bottom) following challenge with 5.6 x 10^4^ TCID_50_ A/FM, for mice infected with RSV-A2 (left panel) or RV1B (right panel) following rAd immunization. n = 8 for B/NP-rAd immunized mice, n = 10 for A/NP+M2-rAd immunized groups. Weight loss data were analyzed on the day indicated by the arrow by One-Way ANOVA using the Holm-Sidak method with the A/NP+M2-rAd group as predetermined control. Significance of differences: P<0.001 A/NP+M2-rAd vs. B/NP-rAd and RSV-A2+B/NP-rAd; P<0.001 A/NP+M2-rAd vs. B/NP-rAd and RV1B+B/NP-rAd. Survival data were analyzed by log-rank test with the Holm-Sidak method for pairwise comparisons. Regardless of prior infection, all groups receiving A/NP+M2-rAd were significantly different (P<0.05) from groups receiving B/NP-rAd, but not from other groups receiving A/NP+M2-rAd.

Human vaccinees might also experience respiratory infections in the interval between immunization with a universal vaccine and a subsequent influenza infection. To model this situation, we studied the impact of intervening infection with RV1B or RSV-A2 following universal vaccine administration but before IAV challenge (Fig 4A). Mice were immunized with A/NP+M2-rAd or B/NP-rAd and then exposed to RSV-A2 or RV1B one month later. Immune responses to RSV-A2 and RV1B were assessed (Fig 4B and 4C). Four weeks after RV1B or RSV-A2 infection, mice were challenged with A/FM. T-cell responses to NP147 in the lung were lower in mice with intervening infection compared to mice given A/NP+M2-rAd only (Fig 4D). Intervening infection with RV1B or RSV-A2 did not affect the outcome of challenge infection. Regardless of intervening infection, mice given universal vaccine lost minimal weight following challenge, and 100% survived (Fig 4E).

## Discussion

Immune history may have an impact on responses to a subsequent infection or immunization. Prior research has extensively described the phenomenon of immune responses to prior infections altering the responses to subsequent infection with a different pathogen [13]. Thus, testing candidate vaccines in animals with previous exposures can provide additional information relevant to human vaccination.

Our study tested the influence of exposure to various respiratory viruses on the performance of a universal vaccine designed to stimulate an immune response to conserved influenza A antigens. We gave respiratory viruses 28 days before immunization (A/Udorn, B/Ann Arbor, RSV-A2, and RV1B), 7 days before immunization (RSV-A2 and RV1B), and 28 days after immunization (RSV-A2 and RV1B). Subtle inhibition of protection might not be detected under the conditions used, because the vaccine alone gave such a high degree of protection (i.e., 100% survival), but no reduction in protection by the universal vaccine was demonstrated for the viral respiratory infections. The experimental conditions used also would have missed enhancement of protection, so the model might underestimate the potential of a vaccine, and in humans the vaccine might outperform expectations derived from naïve animal models. Protection against influenza challenge was maintained not only following respiratory infection one month or one week earlier, but also in the case of intervening infection. In some cases, prior infection enhanced immune responses to the universal vaccine. For example, A/Udorn infection before vaccination boosted recall immune responses to the vaccine (Fig 1D and 1E).

Infections at a longer interval before vaccination might have a different impact than recent infections. Future studies can address longer time intervals, to determine the impact on vaccination at different points in the development of immune memory to a preceding infection. Different pathogens might present different cross-reactive epitopes; we chose examples to study. The impact of infections could also depend on host genetics, and the dose of infecting pathogen or vaccine. Inflammatory effects and tissue damage due to previous infection could also play a role. Future studies can address these points. The findings so far provide encouragement that the NP+M2 vaccine is effective not only in pathogen-free responders, but in the more realistic setting of responders experienced with respiratory infection.

According to standard recommendations, conventional influenza vaccines should not be administered if the intended recipient is ill, but those with recent infection remain eligible [43]. To model vaccination after recent infection, we assessed the performance of A/NP+M2-rAd given 7 days after respiratory infection. As determined by morbidity and survival following challenge, protection afforded by A/NP+M2-rAd was not diminished by respiratory viral infections occurring 7 days before immunization. We also observed comparable serum antibody and lung T-cell responses despite recent infections.

Our findings demonstrate that prior infection with A/Udorn induces cross-protection, also termed heterosubtypic immunity. Heterosubtypic immunity has been described many times in the literature for mice and many other animal species [38], and is likely also induced in humans [44–47]. In this case A/Udorn (H3N2) partially protected against A/FM (H1N1) (40% of the mice survived challenge, Fig 1F). In contrast, A/NP+M2-rAd induced more potent immune responses, which were sufficient to protect all mice.

Antibody responses to M2e are weak and variable in mice following influenza virus infection or immunization with cold-adapted influenza virus [1, 4,48–50]. Similarly, in humans, induction of an M2e-specific antibody response following influenza infection is highly variable [51–53]. In agreement with those findings, an M2e-specific antibody response was not induced by A/Udorn infection followed by B/NP-rAd immunization (Fig 1D). However, focused immunization, for example by M2-rAd, more effectively induces an antibody response. Previous work has shown that immunization strategies using engineered M2 conjugates or expression vectors are effective against influenza challenge [35, 50, 54]. While our previous studies showed that M2-rAd alone provides protection, A/NP+M2-rAd provides protection superior to either component alone [1, 3].

For adenovirus-vectored vaccines and gene therapies, interference by pre-existing immunity to the vector can indeed be a concern [55, 56]. Immunity to adenovirus serotype 5 (Ad5) is prevalent in the human population and can be a barrier to subsequent use of Ad5-based vectors, such as the A/NP+M2-rAd universal vaccine candidate used in this study. One way to overcome this barrier is to use an adenovirus vector to which humans are not exposed. In previous work, we used PanAd3, a nonhuman primate adenoviral vector in the same Ad species C as Ad5 [57], to construct a universal vaccine candidate expressing conserved influenza A antigens NP and M1. Humans have very little or no serum antibody to PanAd3, and a universal vaccine with this backbone protected mice from influenza challenge [58]. Also, mucosal administration of rAd (i.n. or aerosol) appears to circumvent blocking by prior immunity in some cases [59, 60].

The present study focused on viruses causing acute respiratory infections. Chronic infections with viruses, bacteria, and parasites can also have a major influence on host immune responses to subsequent vaccination [15, 25–27]. Given the high rate of pre-existing chronic infection in many parts of the world, their impact on immunizations is of major importance. Expanding upon the cases studied here, future research could examine vaccine performance in models of chronic infection.

Depending on virus and timing of infection, immune responses to the universal vaccine were unchanged, enhanced or modestly reduced compared to responses in mice without prior viral infection. Ultimately, the viral infections we tested did not abrogate protection elicited by the universal vaccine. These results are promising that the vaccine may perform well in humans despite widespread immunity to common respiratory viruses.
